# Short-Term Creep Effect on Strain Transfer from Fiber-Reinforced Polymer Strips to Fiber Bragg Grating-Optical Fiber Sensors

**DOI:** 10.3390/s23031628

**Published:** 2023-02-02

**Authors:** Hai Van Tran, Soo-Yeon Seo

**Affiliations:** Department of Architectural Engineering, Korea National University of Transportation, Chungju 27389, Republic of Korea

**Keywords:** fiber-reinforced polymer strip, optical fiber sensor, fiber Bragg grating, strain transfer, short-term creep effect

## Abstract

In this study, the short-term creep effect (STCE) on strain transfer from fiber-reinforced polymer (FRP) strips to fiber Bragg grating-optical fiber (FBG-OF) sensors was investigated. Thirty OF sensors attached to FRP strips were investigated through three primary test parameters: bond length (40, 60, 80, 100, 120, and 150 mm); adhesive type (epoxy resin, CN adhesive, and epoxy resin combined with CN adhesive); and bonding method (embedded and external bonding methods). The strain transfer ability of the OF sensors was evaluated based on the strain ratio of the OF sensor to the FRP strip under different sustained stresses of 20, 40, 50, and 60% of the FRP ultimate tensile strength (f_u_). From the test results, it was found that the debonding phenomenon occurred at the interface between the FBG-OF sensor and the adhesive and was clearly observed after applying a load for three days. It was also found that the CN adhesive showed better strain transfer compared to the other adhesive types. Regarding the OF sensors bonded by epoxy resin, in order to maintain strain transfer ability under a high level of sustained stress (0.6f_u_), minimum bond lengths of 100 and 120 mm were required for the embedded and external bonding methods, respectively.

## 1. Introduction

At present, fiber-reinforced polymer (FRP) composite is well known as an effective retrofitting material for reinforced concrete members due to its advantages, such as lightweight, good corrosion resistance, compatibility with concrete, and high strength-to-weight ratio [1]. Among installing techniques, the most common method, known as the externally bonded (EB) method, has been applied by attaching FRP sheets or plates to concrete surfaces with epoxy resin. In addition, a novel technique is called the near surface-mounted (NSM) method, in which FRP bars or strips are embedded into grooves on the concrete cover and filled with epoxy resin afterward [2,3]. Compared to the EB method, the NSM method with a full bond length along the FRP composite can more effectively improve the strength capacity of a reinforced concrete (RC) member when it is used for flexural strengthening. Moreover, the deformation capacity can be increased by designing to have a partially debonded region in the NSM method [4,5].

Normally, in retrofitting with FRP composite, epoxy resin is used for bonding it to concrete surfaces because epoxy resin has good stress transfer ability and the capacity for concentrated stress redistribution [6]. However, the strength of epoxy adhesive can be decreased because of construction mistakes and harsh environmental conditions, especially temperature. Therefore, a series of studies has been performed on smart composites in which FRP materials were combined with sensors. By retrofitting these smart composites, the performance of the FRP material and the health condition of the structural elements were monitored by means of strain variation of FRP materials, detection of new cracks, and expansion of cracks [7,8].

Recently, most smart composites have been fabricated with OF sensors based on fiber Bragg grating (FBG) technology [9]. Bragg grating, discovered in 1978 by Ken Hill [10], is based on the optical principle of “total internal reflection” in order to confine and transmit light in the core. Later, the industrial photo-inscription technique for fiber Bragg grating sensors was demonstrated in 1989 by Meltz et al. [11]. FBG sensors have been used mainly in the aeronautical industry due to their small sizes with fast response and immunity to electromagnetic interference. Sensing using OF sensors has attracted the interest of many researchers in the development of structural health monitoring (SHM) systems due to their useful inherent advantages, such as high durability, stability in long-term measurement, and the possibility of making multiple sensors along with a single optical fiber [12,13]. A review article dedicated to the research and development activities of FBG sensors for structural health monitoring was published by Majumder et al. [14].

Thus far, many studies have been performed to evaluate OF sensors for structural health monitoring and assessment of reinforced concrete members. Crack-opening displacements in concrete can be detected and measured using a fiber-optic laser speckle-intensity sensor that was developed by Zhang and Ansari [15,16]. Gu et al. [17] adopted OF sensors for concrete structures in order to establish crack detection methodologies based on monitored performance. Zhao et al. [18] investigated the debonding mechanism of FRP systems with concrete surfaces by OF sensors embedded at their interface. Furthermore, OF sensors have also been combined with FRP materials to fabricate a smart composite for retrofitting RC members. Siwowski et al. [19] applied distributed fiber optic sensors (DFOS) in FRP composites for bridge monitoring. Wood et al. [20] adopted the DFOS system substituted for electrical strain gauges to measure the strain distribution in FRP sheets used to retrofit shear wall structures and indicated that the DFOS system can be used to measure two-dimensional spatial strain with high precision. Wang et al. [21] validated the sensing capacity of OF sensors embedded in FRP bars using the tensile, bond, and beam flexural tests.

In order to reduce the fabrication period and simplify the fabrication procedure, Seo et al. [22,23] performed studies to suggest a minimum bond length between OF sensors and FRP materials through the tensile test of FBG-FRP composite using an analytical approach. However, in these studies, the creep effect on the epoxy resin used as an adhesive between the FBG sensor and the FRP material, which may affect strain transfer, was not considered. According to Tam et al. [24], the strength of epoxy resin can be significantly decreased under highly sustained stresses within a short period of time. So far, few studies have investigated the creep effect on strain transfer from an FRP strip to an OF sensor, especially OF sensors bonded with limited bond length. Therefore, more studies related to the creep effect on the shear transfer of FBG-FRP composite are needed.

The primary objective of this study was to experimentally assess the short-term creep effect of different stress levels on the shear transfer of FBG-FRP composites. The FBG-FRP specimens were fabricated and investigated through three different parameters, namely bond length, bonding method, and adhesive type. In addition, strain values from the FBG sensors were validated with those from electrical strain gauge (ESG) sensors attached at the middle position of the bond length.

## 2. Experimental Program

### 2.1. Materials

**Figure 1:**
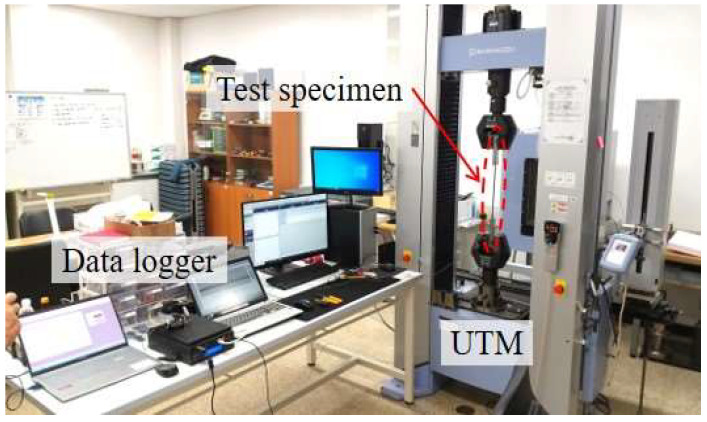
Tensile test set-up for the FRP strip.

**Figure 2:**
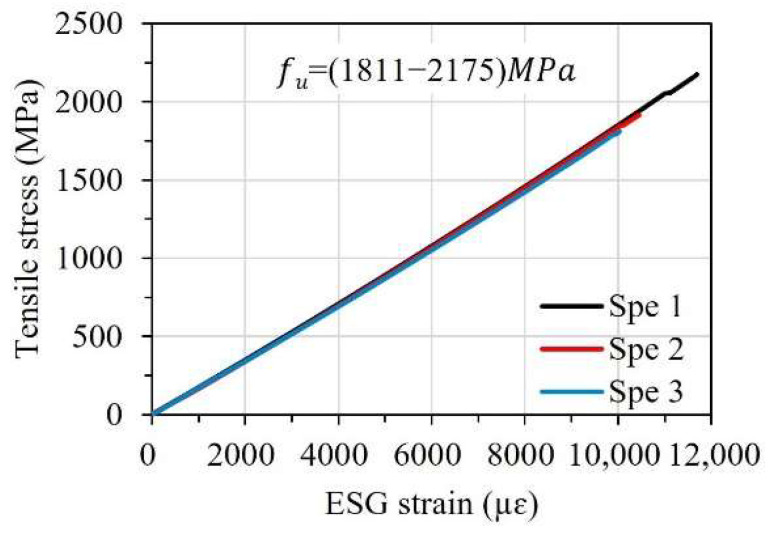
Stress–strain relationship of the FRP strip.

In this study, carbon fiber-reinforced polymer strips (SK-CPS-0512) supplied by the SK Group (Seoul, Republic of Korea) were utilized to manufacture FBG-FRP specimens. Three tensile specimens of FRP strips with the same cross-section dimensions as the FRP strips used for the FBG-FRP composite specimens (a thickness of 1.2 mm and a width of 15 mm) were fabricated and tested in tension in accordance with ASTM D3039 [25]. Figure 1 illustrates the tensile test setup of the FRP strip. The stress–strain relationship and mechanical properties are presented in Figure 2 and Table 1, respectively.

The same type of epoxy resin (SK-CPA10, SK Chemicals Co., Gyeonggi-do, Republic of Korea) used in previous studies by Seo et al. [22,23] was applied as an adhesive. The epoxy resin was mixed with a hardener in a 2:1 ratio in accordance with the manufacturer’s guidelines. The mechanical properties of the epoxy resin provided by the manufacturer are presented in Table 2. The epoxy resin was employed for bonding FBG sensors to the surface of the FRP strips and the two overlapped FRP strips where OF sensors were embedded between the two FRP strips. In addition, cyanoacrylate (CN) adhesive (Tokyo Measuring Instruments Laboratory Co., Tokyo, Japan) was used to bond the FBG sensors to the FRP strips. The CN adhesive is considered an effective adhesive to bond strain gauges with a short curing time of 20–60 s. The attachment of the FBG sensors to the surface of the FRP strips can be accomplished in a short period of time. According to the OF sensor manufacturer, CN adhesive can be applied to bond OF sensors on metallic and plastic surfaces. In a study by Motwani et al. [26], CN adhesive even showed greater effectiveness than epoxy resin when bonding OF sensors to carbon fiber-reinforced polyphenylene sulphide.

**Figure 3:**
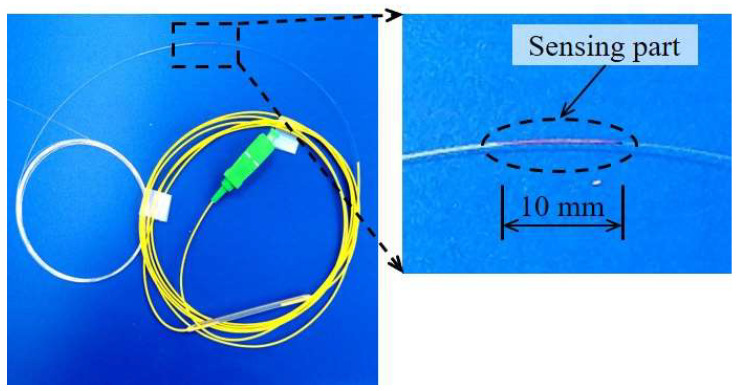
A picture of the FBG-optical fiber sensor.

**Figure 4:**
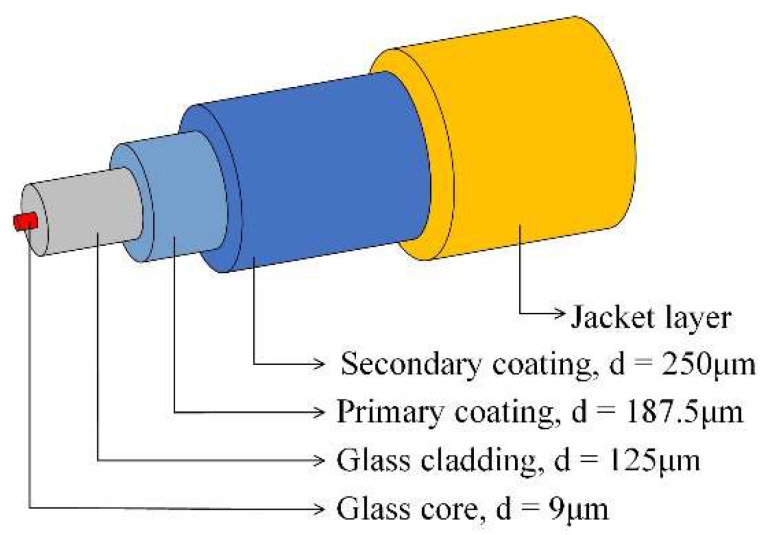
Configuration of the FBG-optical fiber sensor.

To fabricate the FBG-FRP specimens, a fiber Bragg grating-optical fiber sensor (Corning SMF-28, FBG Inc., Seoul, Republic of Korea) with a 10 mm sensing part and diameter of 9 μm was utilized (see Figure 3). The glass core sensing part was covered with glass cladding with a diameter of 125 μm and then coated with primary and secondary coating layers with diameters of 187.5 and 250 μm, respectively (see Figure 4). The primary and secondary coating layers were made of polymethyl methacrylate (PMMA) material. Finally, the entire length of the FBG sensor was covered with a jacket layer to avoid any damage due to the brittle characteristics of the glass core part. The coating surface was directly bonded to the epoxy resin after removing the jacket layer.

### 2.2. Test Parameters

To investigate the short-term creep effect on strain transfer from the FRP strips to the OF sensors, 30 OF sensors in total bonded to FRP strips were investigated through three parameters (i.e., bond length, bonding method, and adhesive type). In detail, bond lengths of 40, 60, 80, 100, 120, and 150 mm, two bonding methods of embedded and external bonding, and three types of adhesive (epoxy resin, CN adhesive, and epoxy resin combined with CN adhesive) were studied. Table 3 shows the test parameters of the OF sensors bonded to the FRP strip of each specimen. The “X-Y-Z” format was adopted as abbreviated names to classify the 30 OF sensors: “X” denotes the adhesive type, “Y” denotes the bonding method, and “Z” denotes the bond length. For example, E-EM-80 indicates that the OF sensor was bonded with epoxy resin adhesive following the embedded method with a bond length of 80 mm. It should be noted that the OF sensors of the E-EBM-80b and EC-EBM-120 cases were damaged during fabrication; therefore, no data were collected from these cases.

### 2.3. Fabrication of FBG-FRP Specimens

**Figure 5:**
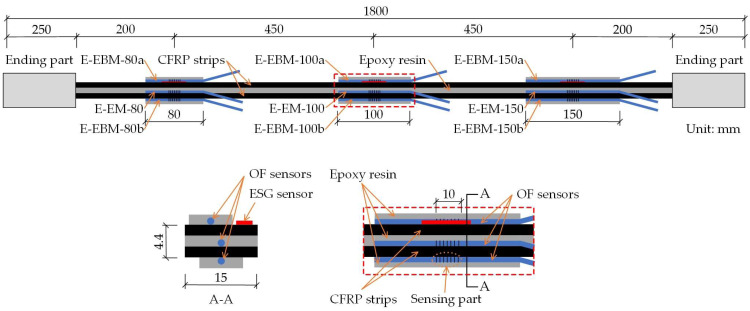
Geometrical details of the E specimen.

**Figure 6:**
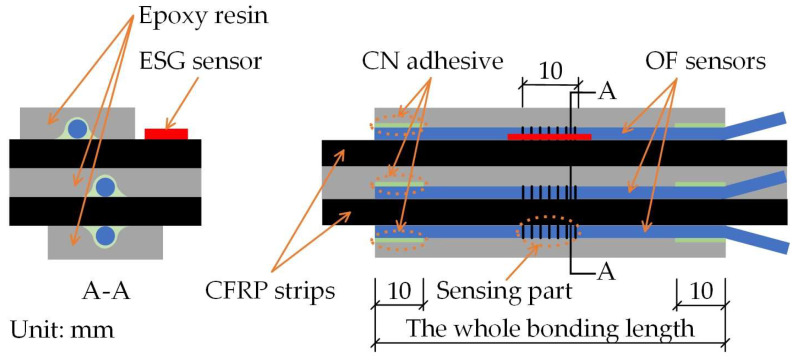
Illustration of the FBG-FRP segment of the EC specimen.

**Figure 7:**
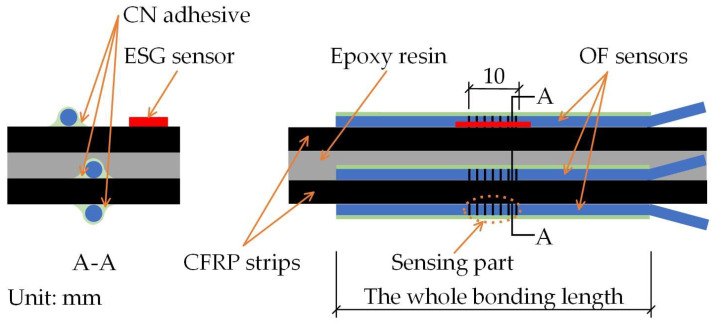
Illustration of the FBG-FRP segment of the C specimen.

Figure 5 shows the geometrical details of the E specimen. The E specimen was made by combing two FRP strips with widths of 15 mm, thicknesses of 1.2 mm, and lengths of 1800 mm. Between the two FRP strips bonded with epoxy resin, OF sensors were attached at three positions with different bond lengths using the embedded bonding method. In addition, OF sensors were attached to both surfaces using the external bonding method with different bond lengths. The EC and C specimens had similar geometrical details to the E specimens; details of the OF sensor locations can be seen in Figure 6 and Figure 7, which illustrate the FBG-FRP segments for the EC and C specimens, respectively. To obtain sufficient data to determine the minimum bonding length between the OF sensor and the FRP strip, an additional specimen, A, was prepared. The A specimen was fabricated with the same overall geometrical dimensions as the other specimens except for the adhesive and bond length for externally bonded OF sensors. In detail, three types of adhesive were considered for the externally bonded OF sensors: epoxy resin only, epoxy resin and CN adhesive combined, and CN adhesive only. The bond lengths of the OF sensors were 120 mm for the first and second cases and 100 mm for the third case.

**Figure 8:**
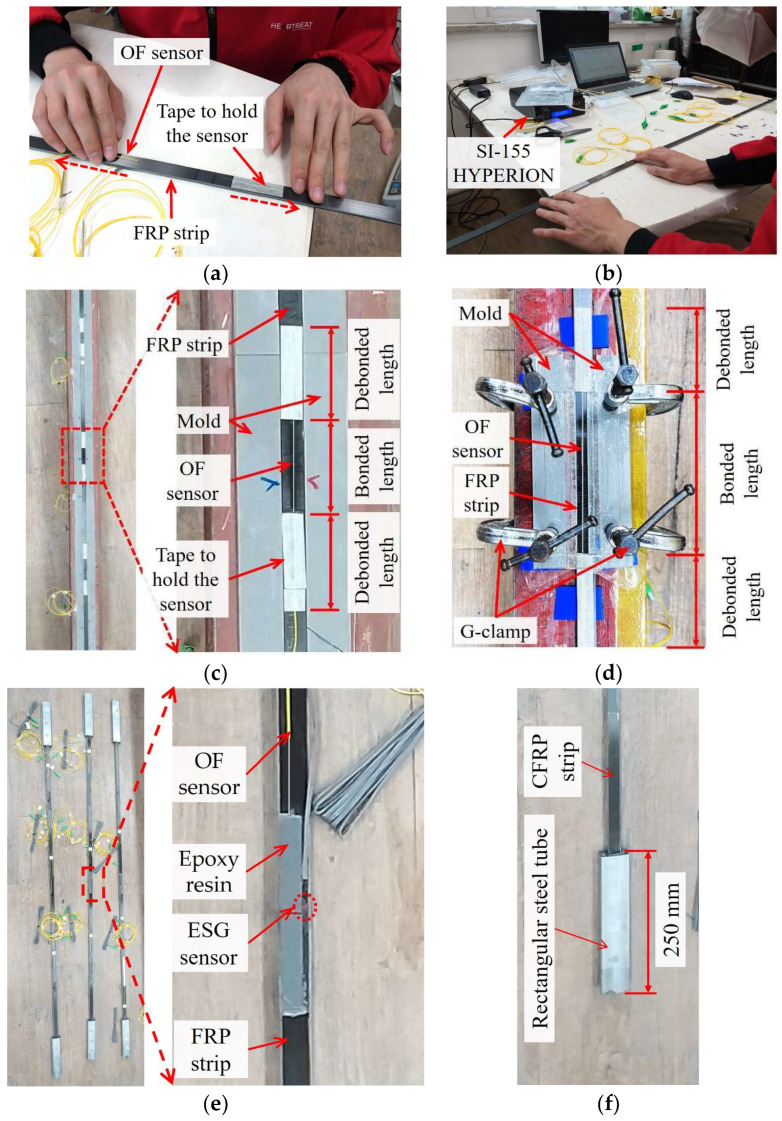
Fabrication process of FBG-FRP composite: (**a**) Applying a small prestress on the FBG-OF sensor; (**b**) Controlling the small prestress; (**c**) Embedded bonding method; (**d**) External bonding method; (**e**) FBG-FRP test specimens; (**f**) Ending part.

Figure 8 shows the fabricating process of the FBG-FRP specimens in the laboratory. The surfaces of each FRP strip were roughened using emery paper to increase the adhesion between substrate and adhesive. Next, they were cleaned with acetone solution before attaching the OF sensors. The OF sensors were fixed using adhesive tape after applying a small amount of pressure, as shown in Figure 8a. This small pressure corresponding to 5% of the strain capacity of the OF sensor was applied in order to keep the OF sensor straight during the fabrication process. The magnitude of the applied prestress can be controlled through the shift wavelength variation of the OF sensor, which was observed on the HYPERION SI-155 optical sensing instrument (see Figure 8b). Then, the FRP strip attached with the OF sensors was placed in a mold and filled with epoxy resin to join it with the other FRP strip (see Figure 8c). Regarding the embedded OF sensors, the adhesive tapes were kept when filling with epoxy resin to achieve the desired bond length. The specimen was demolded after curing for 24 h in order to attach other OF sensors to the surface of the FRP strips.

After embedding three OF sensors between two FRP strips, six OF sensors were externally bonded to the front and back surfaces of the FRP strips (see Figure 8d). The externally bonded OF sensors were fabricated by following the same procedure as the embedded OF sensors. Then, to evaluate strain transfer from the FRP strip to the OF sensors, ESG sensors were attached at the middle position of the bond length (see Figure 8e). To ensure the complete transfer of tensile stresses, the ending parts of the test specimens were bonded into rectangular steel tubes using epoxy, as shown in Figure 8f. The length of the FRP strip embedded inside the rectangular steel tube was calculated in order to prevent debonding, which can happen before fracture failure of the FRP strip. Finally, the FBG-FRP specimens were cured for 7 days under temperature conditions of 23 ± 3 °C in accordance with ASTM 3039 [25] before performing the experiment. Regarding the EC specimen, after using adhesive tape to maintain the small pressure, CN adhesive was used to bond the OF sensors to the FRP strip with a bond length of 10 mm at each ending part. The remaining part of the entire bond length was bonded using epoxy resin (see Figure 6).

### 2.4. Short-Term Creep Test

**Figure 9:**
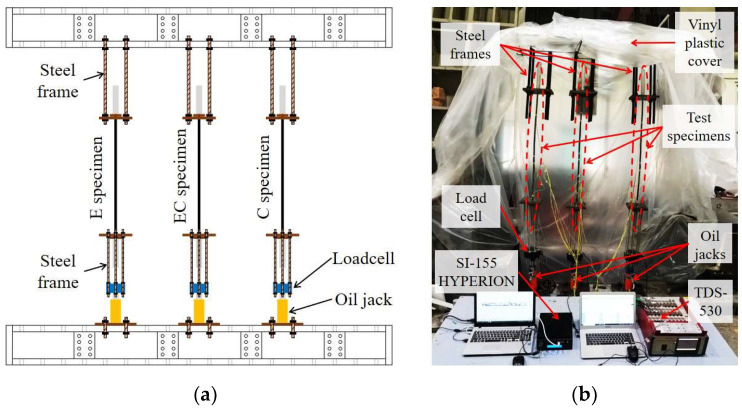
The short-term tensile test setup: (**a**) Setup schema; (**b**) Picture of the test setup.

The setup schema of the short-term tensile test is shown in Figure 9a. One ending part of the test specimen was fixed by the steel frame, while the other was subjected to sustained tensile loads using oil jacks (TECPOS TDC-2015) with a capacity of 200 kN. The applied load was controlled using a load cell (CAS LS-20B) with a capacity of 200 kN. Meanwhile, the SI-155 HYPERION optical sensing instrument and the TDS-530 data logger were used to record the data from the FBG and ESG sensors, respectively (see Figure 9b).

**Figure 10:**
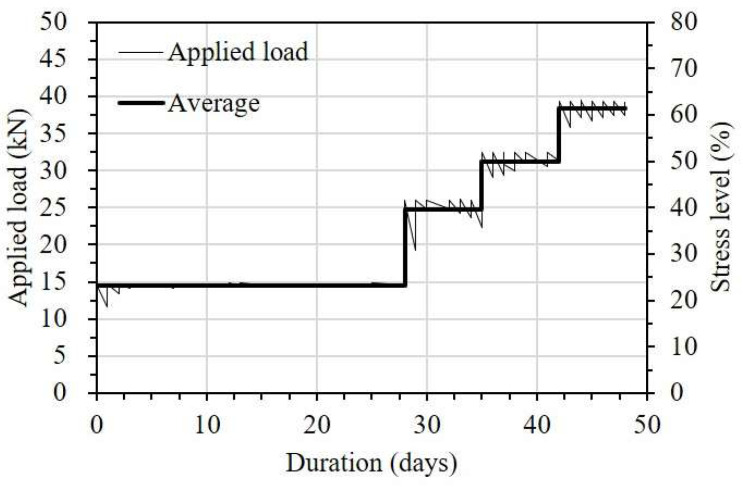
Loading history for the short-term creep test.

According to ACI 440 [27], the total applied stress for design purposes is limited to approximately 55% of the ultimate tensile strength in CFRP in order to prevent creep rupture failure. At present, a limited number of experimental results have been reported regarding the minimum bond lengths needed to maintain the strain transfer ability from FRP strips to OF sensors corresponding to differently sustained stresses. Therefore, test specimens were subjected to four different stress levels of 20, 40, 50, and 60%, corresponding to the ultimate tensile strength (f_u_) of the FRP strip. In the progression of stress, a stress level of 0.2f_u_ was maintained for the first 4 weeks, and then the next increments were maintained for 1 week each. Figure 10 illustrates the loading history applied to the test specimens during the short-term creep test. The applied load was checked and controlled every day to maintain the target load before recording the data. The vinyl plastic cover was used to create a small and enclosed space around the testing area to control temperature and humidity (see Figure 9b). During the test, the temperature and humidity conditions were maintained within the ranges of 23 ± 3 °C and 50 ± 10%, respectively, in accordance with ASTM 3039 [25].

## 3. Test Results

### 3.1. Creep Effect on the FRP Strip

**Figure 11:**
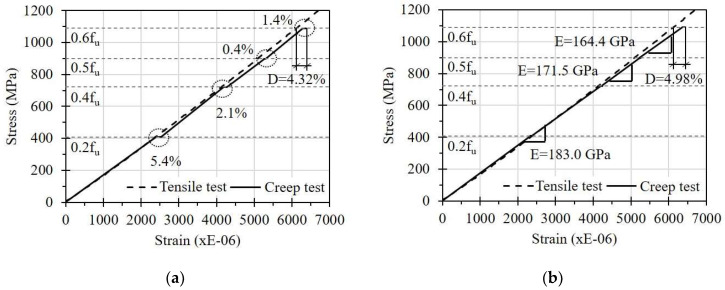
Creep effect on the FRP strip: (**a**) The E specimen; (**b**) The C specimen.

Figure 11 shows the stress–strain curves of the FRP strips under the short-term creep test. The dotted lines in the figures are for comparison, and the stress–strain curves of the FRP strips obtained from a simple tensile test are shown in Figure 2. The solid line shows the relationship between stress and strain of the FRP strip subjected to loads with a duration of 28 days for 0.2f_u_ and 7 days for each subsequent increments. The stresses were calculated by dividing the applied load on the FRP strip by the cross-sectional area of the FRP strip. It should be noted that the displayed strains are the average values of the three strain gauges attached to each FRP strip.

From the graphs, it can be seen that the FRP strip was affected by sustained stress. In detail, the strain on the FRP strip in the E specimen increased by 5.4% due to the creep effect after applying a sustained stress of 0.2f_u_ for 28 days (see Figure 11a). In addition, strain increases due to creep effects were observed after loading for 7 days each at 0.4f_u_ and 0.6f_u_. The additional creep strain generated from the sustained stress at 0.4f_u_ became plastic deformation. Compared with the stress–strain curve of the simple tensile test, the accumulated additional plastic strain due to creep at 0.6f_u_ was 4.32% of the total strain. This implies that the FRP strip may show additional creep strain, even under low sustained stress, and this can be a plastic strain. Similar results were observed on the FRP strip in the EC specimen.

The FRP strip in the C specimen did not show a clear increase in strain under sustained low stresses (see Figure 11b). However, the elastic modulus of the FRP strip decreased gradually under high sustained stress levels. In detail, the elastic modulus of 183 GPa was maintained after applying sustained stress up to 0.4f_u_. However, it decreased to 171.5 and 164.4 GPa after applying stresses of 0.5f_u_ and 0.6f_u_ for 7 days, respectively. As shown in Figure 5, Figure 6 and Figure 7, the locations where the ESG sensors were attached were right next to the adhesives, such as epoxy or CN, to install the OF. Therefore, there is a possibility that the adhesives may have contributed to the difference in creep effect observed on these FRP strips. When the CN adhesive was applied, the creep strain of the FRP strip due to a sustained load at a low stress of 0.2f_u_ was not remarkable. At a high stress of 0.6f_u_, however, the additionally increased plastic strain was 4.98%, which is compatible with that of the E specimen with epoxy resin as the adhesive. From this, it is recommended to consider an additional strain increase of at least 5% due to creep effect when the FRP strip is exposed to sustained tensile stress conditions.

**Figure 12:**
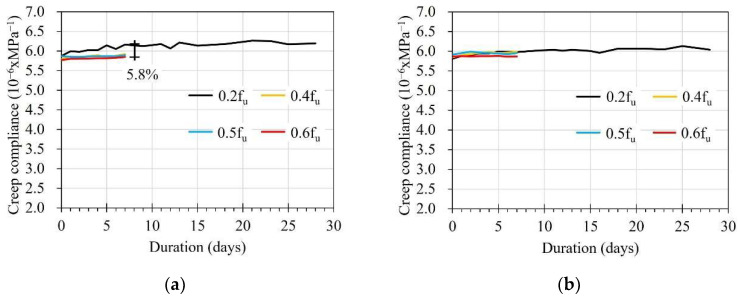
Creep compliance of the FRP strip: (**a**) The E specimen; (**b**) The C specimen.

Table 4 summarizes the creep compliances of the FRP strip, calculated according to:

(1) $$J_{c}(t)=\frac{\varepsilon (t)}{\sigma_{0}}$$

where $J_{c}(t)$ and ε(t) are the creep compliances and strain at time (*t*), respectively, and $\sigma_{0}$ is the applied stress. The applied stresses were calculated by dividing the applied load by the cross-sectional area of the FRP strip. Figure 12a presents the average creep compliances of the FRP strip in the E specimen under different stress levels. The average creep compliance showed a gradual increase over time at 0.2f_u_. After increasing the stress to 0.4f_u_, 0.5f_u_, and 0.6f_u_, it was found that changes in the average creep compliances over time were not significant. It can be seen that a maximum difference of 5.8% was observed between the average creep compliances at 0.2f_u_ after applying the load for 7 days compared to the others. This can be explained by the significantly higher creep strain in the early stage of duration. Meanwhile, no differences between average creep compliances under different stress levels were observed in the C specimen, as shown in Figure 12b. This can be attributed to the strain on the FRP strip due to the creep effect not being displayed clearly in the C specimen (see Figure 11b).

### 3.2. Short-Term Creep Effect on the Sensing Capacity of the OF Sensor

The strain was obtained by converting the reflected wavelength of the OF sensors measured by the SI-155 HYPERION optical sensing instrument, using Equation (2).

(2) $$\Delta \varepsilon =\frac{1}{1-P_{e}}\left[\frac{\Delta \lambda_{B}}{\lambda_{B}}-(\alpha +\xi )\Delta T\right]$$

where $P_{e}=0.22$ is the photoelastic modulus,$\lambda_{B}$ is the reflected wave length, $\alpha =0.55\times 10^{-6}$ is the expansion coefficient corresponding to temperature, and $\xi =8.6\times 10^{-6}$ is the coefficient considering the change of reflection corresponding to temperature [28]. The transferred strain percentages (TSPs) from the FRP strip to the FBG-OF sensors are summarized in Table 5. The TSP values at the start and end points of each duration were calculated at the moments after applying the load and before increasing to a higher load.

#### 3.2.1. Influence of Bonding Methods

**Figure 13:**
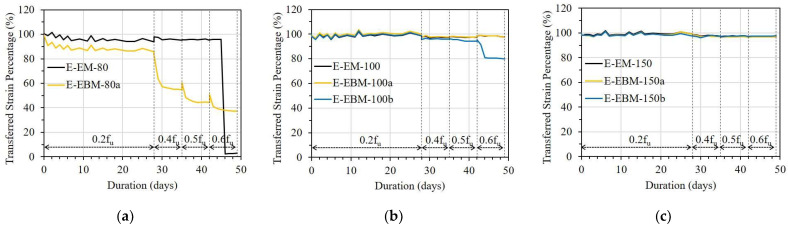
Short-term creep effect on strain transfer of the E specimen with different bonding methods: (**a**) L = 80 mm; (**b**) L = 100 mm; and (**c**) L = 150 mm.

**Figure 14:**
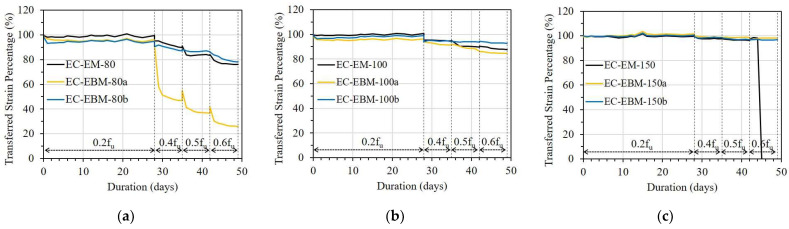
Short-term creep effect on strain transfer in the EC specimen with different bonding methods: (**a**) L = 80 mm; (**b**) L = 100 mm; and (**c**) L = 150 mm.

**Figure 15:**
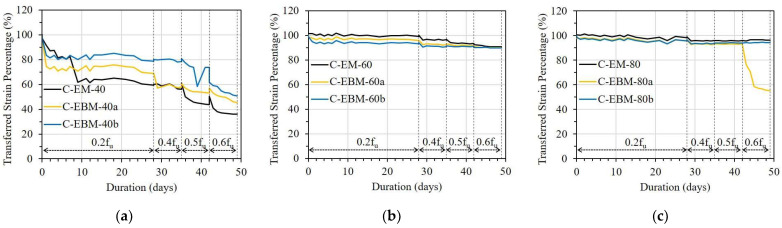
Short-term creep effect on strain transfer of the C specimen with different bonding methods: (**a**) L = 40 mm; (**b**) L = 60 mm; and (**c**) L = 80 mm.

Figure 13, Figure 14 and Figure 15 show the variation in TSPs from the FRP strip to the OF sensors under different stress levels. It can be seen that the bonding methods, namely the embedded method (EM) and the externally bonded method (EBM), affected the strain transfer from the FRP strip to the OF sensors. E-EM-80 showed an overall constant TSP after a small decrement until debonding occurred, whereas E-EBM-80a showed a gradual decreasing pattern over time (see Figure 13a). In detail, the instantaneous TSP values of E-EBM-80a and E-EM-80 were 99% and 100% after applying 0.2f_u_ and then slightly decreased to 86% and 94%, respectively. However, the TSP of E-EBM-80a decreased more rapidly at a stress level of 0.4f_u_, and this decreasing pattern continued corresponding to increasing time. Conversely, E-EM-80 maintained a stable TSP of approximately 94% after the stress level was increased to 0.4f_u_. Even after the stress level was increased to 0.6f_u_, E-EM-80 did not show any decrement in TSP, but the TSP momentarily became “0” on the fourth day. From Figure 13b,c, it can be seen that when the bond length of the OF sensor by the embedded method was 100 mm or more, the strain could be transferred to the OF sensor very effectively for a loading period of 50 days in total. On the contrary, in the case of the external bonding method, it can be seen that the transmission rate of the strain started to decrease at a stress level of 0.4f_u_ at a bond length of 100 mm, and in the case of 150 mm, it was transmitted very effectively throughout the entire period.

In Figure 14, the TSPs of the EM and EBM in the EC specimen are compared. There was a small drop in TSP in the case of the EBM with a bond length of 80 mm 1 day after applying a load of 0.2f_u_, and it was maintained for 28 days before the load level was increased. However, when the load increased to 0.4f_u_, the TSP of one OF sensor abruptly dropped, and the other showed a gradually decreasing pattern. In the case of the EM with a bond length of 80 mm, a small drop in the early days did not occur, but a gradually decreasing pattern was observed when the load was increased to 0.4f_u_. In the case when the bond length was 100 mm, as shown in Figure 14b, there was no significant drop in the TSP of the two bonding methods. However, a small drop and decreasing pattern of TSP occurred at 0.4f_u_ in both bonding methods. This reduction in TSP did not occur in cases where the bond length was 150 mm for both bonding methods (Figure 14c) before 0.6f_u_. However, a rapid drop in TSP occurred in the EM due to bond failure after the stress was increased to 0.6f_u_.

Figure 15 shows the TSP of the OF sensors in the C specimen. In the case of a bond length of 40 mm, regardless of the bonding method, the TSPs of the OF sensors decreased gradually immediately after 0.2f_u_ stress was applied, reaching less than 80% on the 28th day. For the longer bond lengths, the EM showed a slightly better TSP than the EBM during the creep test. In particular, in the case of the embedded method, more than 95% of the TSP can be obtained at 0.6f_u_, even with a bond length of 80 mm (see Table 5).

Regarding the OF sensors bonded with epoxy resin or epoxy resin combined with CN adhesive, the EM showed better efficiency compared to the EBM in the case of the OF sensors having a bond length of 80 mm. According to the theoretical approaches of Ansari et al. and Seo et al. [22,23,29], the shear transfer coefficient from the FRP strips to the embedded OF sensors can be increased due to shear transfer from the FRP strips on both sides compared to the OF sensors bonded externally on one side. However, when the bond length was 150 mm, the TSP of the OF sensor in both the EM and EBM showed more than 97%, and no difference was observed between them.

The TSP can be considered the sensing capability of the OF sensor, which shows the accuracy level for the strain transfer capability from the FRP strip to the OF sensor. From the studies of Seo et al. [22,23], the strain can be transferred sufficiently from the FRP strip to the OF sensor with a bond length of 40 mm if not exposed to sustained loading conditions. When evaluating the sensing capacity of OF sensors bonded to an FRP strip with a bond length not less than 40 mm, the test result indicated a deviation range of 5%. This deviation range is compatible with that of the Omega strain gauges series (Omega Engineering, Inc., Seoul, Republic of Korea). Moreover, when using epoxy or CN adhesive, as in this study, if the bond length is increased in consideration of the creep effect, the sensing capacity of the OF can be improved within the deviation range of 3%.

#### 3.2.2. Influence of Bond Lengths

**Figure 16:**
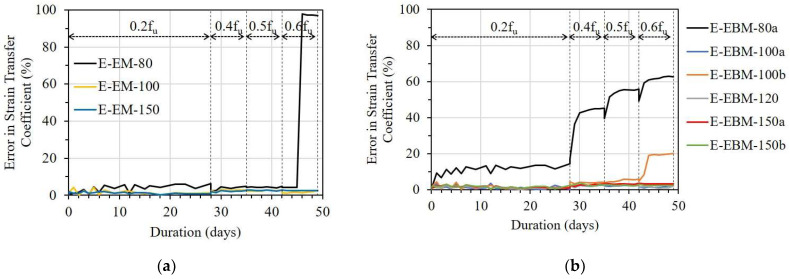
The error in strain transfer coefficient of the E specimen with different bond lengths: (**a**) Embedded method and (**b**) Externally bonded method.

**Figure 17:**
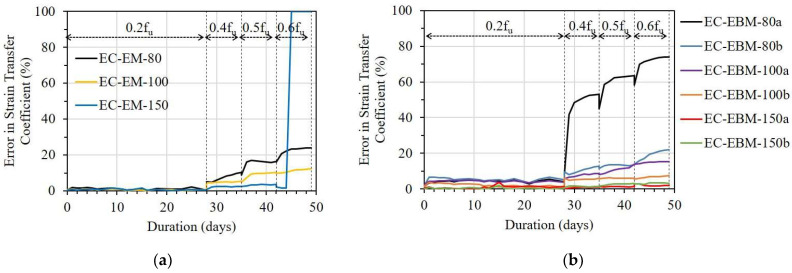
The error in strain transfer coefficient of the EC specimen with different bond lengths: (**a**) Embedded method and (**b**) Externally bonded method.

**Figure 18:**
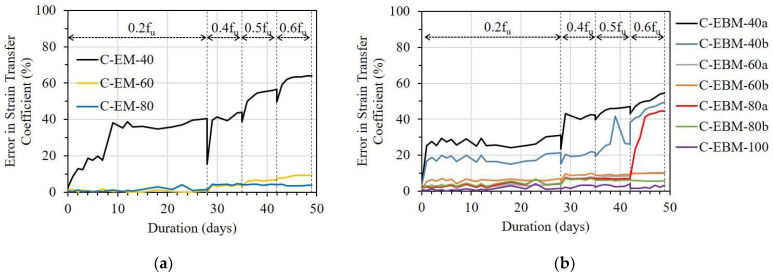
The error in strain transfer coefficient of the C specimen with different bond lengths: (**a**) Embedded method and (**b**) Externally bonded method.

Figure 16, Figure 17 and Figure 18 show errors in the strain transfer coefficient (ESTC) of sensors with different bond lengths on strain transfer from the FRP strip to the OF sensor under sustained stresses. The effect of bond length was assessed through the ESTE values calculated by dividing the decrement of the OF sensor strain value by the corresponding ESG strain value.

Figure 16 presents the error ratio in the ESTC of the E specimen corresponding to bond length. In the case of the EM, E-EM-80 showed a critically sharp increment in ESTC under a stress level of 0.6f_u_; meanwhile, E-EM-100 and E-EM-150 did not show any noticeable changes (Figure 16a). Regarding the case of EBM, the ESTCs of E-EBM-80a and E-EBM-100b were significantly increased from the stress levels of 0.4f_u_ and 0.6f_u_, respectively (Figure 16b). This implies that minimum bond lengths of 100 and 120 mm are recommended for the EM and EBM, respectively, in order to sufficiently transfer the strain from the FRP strip to the OF sensor.

From Figure 17 presenting the ESTC of the EC specimen, in the case of the EM, it can be observed that EC-EM-80 and EC-EM-100 showed high ESTC values of 24 and 12%, respectively, under the stress level of 0.6f_u_. Meanwhile, EC-EM-150 lost its strain transfer ability (see Figure 17a). Similar results can be observed in the case of the EBM, in which the OF sensors with bond lengths of 80 and 100 mm showed high ESTC values of over 70 and 20%, respectively, under a stress level of 0.6f_u_ (see Figure 17b).

According to Motwani et al. [26], CN adhesive with low viscosity (~20 cP at room temperature) may show different stiffness compared to epoxy resin with high viscosity (~25,000 cP at room temperature). Moreover, the poor water resistance of CN adhesive may cause a change in the chemical bond, leading to a reduction in bonding strength [26]. Conversely, epoxy resin has good water resistance. Because of the incompatibility between the epoxy resin and the CN adhesive, the strain transfer ability of the OF sensor can be decreased. Therefore, EC-EM-150 lost its strain transfer ability, even with a longer bond length compared with EC-EM-80 and EC-EM-100. From the result, the combination of epoxy resin and CN adhesive is not recommended for attaching OF sensors to FRP strips.

Figure 18 shows the influence of bond lengths on the ESTC of the C specimen. For the OF sensors bonded by the EM, the E-EM-40 showed a serious ESTC of 40%, even under a stress level of 0.2f_u_. With longer bond lengths, however, the ESTC values of C-EM-60 and C-EM-80 were maintained at 10 and 5%, respectively, under a stress level of 0.6f_u_ (see Figure 18a). Regarding the OF sensors bonded by the EBM, a minimum bond length of 100 mm was necessary to adequately transfer the strain from the FRP strip to the OF sensor (see Figure 18b).

#### 3.2.3. Influence of Different Types of Adhesives

**Figure 19:**
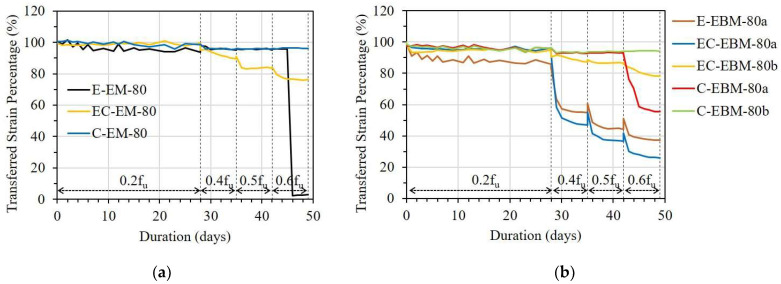
Short-term creep effect on shear transfer with different types of adhesives: (**a**) EM with bond length of 80 mm and (**b**) EBM with bond length of 80 mm.

**Figure 20:**
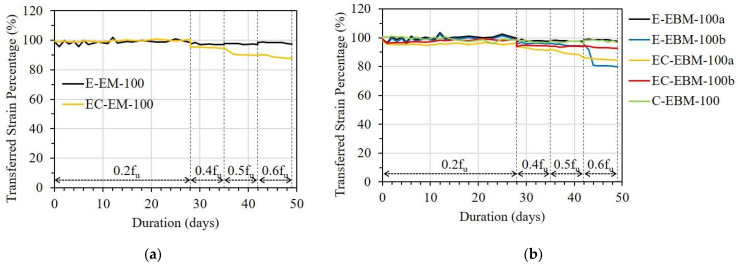
Short-term creep effect on shear transfer with different types of adhesives: (**a**) EM with bond length of 100 mm and (**b**) EBM with bond length of 100 mm.

Figure 19 and Figure 20 present the effect of adhesive type on the TSP from the FRP strip to the OF sensor with bond lengths of 80 and 100 mm, respectively. With a bond length of 80 mm in both cases of the EM and EBM, it can be seen that the OF sensor bonded by CN adhesive showed the best TSP; meanwhile, the OF sensor bonded by epoxy resin combined with CN adhesive showed the worst TSP (see Figure 19). The effectiveness of CN adhesive can be observed clearly under sustained stress from 0.4f_u_.

Under sustained stress from 0.4f_u_, the TSP of the OF sensor bonded with epoxy resin could be significantly decreased due to debonding when the bond length was insufficient to maintain its strain transfer ability. According to the comparison between different adhesive types, it is believed that the debonding occurred at the interface between the OF sensor and the adhesive. In this case, the debonding occurred at the interface between the secondary coating layer of the OF sensor and the epoxy resin.

Regarding the longer bond length of 100 mm, the difference in the TSP caused by the various adhesive types decreased. In detail, in the case of the EM, the OF sensor bonded by epoxy resin showed a TSP 8% higher than the OF sensor bonded by epoxy resin combined with CN adhesive (see Figure 20a). Meanwhile, the OF sensor bonded by CN adhesive revealed a TSP that was approximately 17% higher than the OF sensor bonded by the other type of adhesive, which showed the worst TSP (see Figure 20b).

#### 3.2.4. Scanning Electron Microscope

In order to obtain further insight into the interfaces between OF sensors and epoxy resin, as well as the component layers of the OF sensors, a scanning electron microscope (SEM) analysis was performed after finishing the creep test. The cross-section samples were prepared using a Cross Section Polisher (IB-19510CP, JEOL Ltd., Tokyo, Japan). Then, a Field Emission Scanning Electron Microscope (JSM-7610, JEOL Ltd., Japan) was used for the SEM analysis.

**Figure 21:**
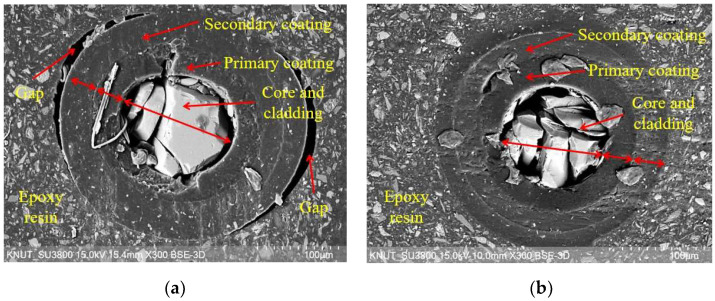
Scanning electron microscope images of FBG sensors in the E specimen: (**a**) Bond length of 80 mm and (**b**) Bond length of 150 mm.

Figure 21a,b show the SEM images of the OF sensors in the E specimen with bond lengths of 80 and 150 representing the cases with and without debonding, respectively. It can be seen that the core and cladding parts were broken after the preparation procedure of the samples due to their brittle characteristics. Meanwhile, the primary and secondary coatings were well preserved. Figure 21a shows the SEM image of E-EBM-80a, which showed a severe reduction in TSP due to debonding. It can be seen that clear gaps appeared due to debonding. Meanwhile, no gaps were observed between the component layers of the OF sensor, such as the primary and secondary coatings or the primary coating and core. This is consistent with the assumption that the bond strength between these layers is higher due to the long bond length compared to the bond strength between the OF sensor and the epoxy layer. In contrast, no gaps were observed in the SEM image of E-EBM-150a due to an unremarkable reduction in TSP (see Figure 21b).

## 4. Conclusions

In this study, the short-term creep effect on strain transfer from the FRP strip to the FBG-OF sensor was investigated under sustained stress levels of 20, 40, 50, and 60% of the ultimate tensile strength of the FRP strip. The three main investigated parameters were bond length, adhesive type, and bonding method. From the test results, the main conclusions were obtained as follows:

(1) According to previous studies regarding the FBG-OF sensor bonded by epoxy resin, it was found that a bond length of 40 mm was sufficient to control the shear lag effect for simple tensile loading. However, under short-term creep conditions to high sustained stress, such as 60% of the ultimate strength of the FRP strip, to maintain a strain carrying capacity of more than 95%, minimum bond lengths of 100 mm and 120 mm are required for the embedded and external bonding methods, respectively.
(2) Regarding the OF sensor bonded with epoxy resin, when the bonding length was 80 mm or less, the strain transferred from the FRP strip to the OF sensor can be greatly reduced, even at stress levels of 40% of the ultimate strength of the FRP strip. This is due to adhesion damage at the interface between the secondary coating of the OF sensor and the adhesive; this phenomenon can be observed at the early stage of the loading duration.
(3) The OF sensor bonded with CN adhesive had better ability in strain transfer compared with the other adhesive types with the same bond length. In particular, in the case of the embedded method with a bond length of 80 mm, more than 95% of the TSP can be obtained at a stress level of 60% of the ultimate strength of the FRP strip.
(4) Regarding the OF sensor bonded with epoxy resin combined with CN adhesive, it is not recommended due to incompatibility in the mechanical properties.
(5) Sustained stress can cause additional creep deformation of FRP strips, even at low stress levels. Furthermore, it is recommended that an additional strain increase of at least 5% be considered due to creep effect when the FRP strip is exposed to conditions of sustained tensile stress.

## Figures and Tables

**Table 1:** Dimension and mechanical properties of the FRP strip.

Type	Thickness (mm)	Width (mm)	Tensile Strength (MPa)	Elastic Module (MPa)
SK-CPS-0512 (CFRP strip)	1.2	15	1813	183,000

**Table 2:** Mechanical properties of epoxy resin.

Type	Compressive Strength (MPa)	Shear Bond Strength (MPa)	Bond Strength to Concrete (MPa)
SK-CPA10	90	10	1.5

Data were provided by the manufacturer.

**Table 3:** List of parameters of FBG-optical fiber sensors bonded on FRP strips.

Specimen Name	Name of FBG-OF Sensor	Bond Methods	Adhesive Types	Bond Length (mm)
E specimen	E-EBM-80a	External bond	Epoxy resin	80
	E-EBM-80b ^(1)^			80
	E-EBM-100a			100
	E-EBM-100b			100
	E-EBM-150a			150
	E-EBM-150b			150
	E-EM-80	Embedded bond		80
	E-EM-100			100
	E-EM-150			150
EC specimen	EC-EBM-80a	External bond	Epoxy resin and CN adhesive	80
	EC-EBM-80b			80
	EC-EBM-100a			100
	EC-EBM-100b			100
	EC-EBM-150a			150
	EC-EBM-150b			150
	EC-EM-80	Embedded bond		80
	EC-EM-100			100
	EC-EM-150			150
C specimen	C-EBM-40a	External bond	CN adhesive	40
	C-EBM-40b			40
	C-EBM-60a			60
	C-EBM-60b			60
	C-EBM-80a			80
	C-EBM-80b			80
	C-EM-40	Embedded bond		40
	C-EM-60			60
	C-EM-80			80
A specimen	E-EBM-120	External bond	Epoxy resin	120
	EC-EBM-120 ^(1)^		Epoxy resin and CN adhesive	120
	C-EBM-100		CN adhesive	100

^(1)^ The FBG-OF sensor was broken during fabrication.

**Table 4:** Creep compliance of the FRP strip.

Applied Stress	Creep Compliances (10^−6^ × MPa^−1^)									COV (%)		J_c,end_/J_c,start_ (%)
	Specimen Name	ESG-1		ESG-2		ESG-3		Average				
		Start ^(1)^	End ^(2)^	Start	End	Start	End	Start	End	Start	End	
0.2f_u_	E specimen	5.85	6.48	5.70	5.84	6.08	6.26	5.88	6.19	3.27	5.26	105.27
	EC specimen	5.73	6.22	5.88	6.05	5.82	6.07	5.81	6.11	1.29	1.50	105.16
	C specimen	5.82	6.33	5.65	5.81	5.73	5.91	5.73	6.02	1.46	4.59	105.06
0.4f_u_	E specimen	5.87	5.97	5.68	5.77	5.84	6.01	5.80	5.92	1.77	2.18	102.07
	EC specimen	5.88	6.08	5.92	6.02	5.74	5.89	5.84	6.00	1.65	1.58	102.74
	C specimen	5.79	5.95	5.77	5.80	5.72	5.94	5.76	5.90	0.67	1.38	102.43
0.5f_u_	E specimen	5.91	5.96	5.72	5.75	5.97	5.97	5.87	5.89	2.27	2.17	100.34
	EC specimen	5.96	5.99	5.93	5.95	5.84	5.91	5.91	5.95	1.05	0.71	100.68
	C specimen	5.79	5.86	5.78	5.91	5.71	5.82	5.76	5.86	0.76	0.77	101.74
0.6f_u_	E specimen	5.86	5.87	5.59	5.74	5.85	5.94	5.77	5.85	2.66	1.74	101.39
	EC specimen	5.91	5.94	5.77	5.92	5.83	5.95	5.84	5.93	1.23	0.29	101.54
	C specimen	5.83	6.09	5.84	5.91	5.87	5.90	5.85	5.97	0.41	1.80	102.05

^(1)^ Instantaneous creep compliance after applying load; ^(2)^ Creep compliance value before increasing load.

**Table 5:** Transferred strain percentages from the FRP strip to the FBG-optical fiber sensors.

Specimen Name	Name of FBG-OF Sensor	Transferred Strain Percentage at Stress Level of (%)							
		0.2f_u_		0.4f_u_		0.5f_u_		0.6f_u_	
		Start ^(1)^	End ^(2)^	Start	End	Start	End	Start	End
E specimen	E-EBM-80a	99	86	83	55	61	44	51	37
	E-EBM-80b ^(3)^	-	-	-	-	-	-	-	-
	E-EBM-100a	99	100	97	98	98	98	99	98
	E-EBM-100b	98	99	96	96	96	94	95	80
	E-EBM-150a	99	99	98	97	96	97	96	97
	E-EBM-150b	98	98	97	97	97	97	97	98
	E-EM-80	100	94	98	95	96	95	96	3
	E-EM-100	99	99	98	97	98	97	99	98
	E-EM-150	98	99	98	98	97	97	97	97
EC specimen	EC-EBM-80a	99	96	92	47	55	37	42	26
	EC-EBM-80b	98	95	90	87	89	86	86	78
	EC-EBM-100a	98	96	94	91	92	86	86	85
	EC-EBM-100b	99	99	94	94	94	94	94	93
	EC-EBM-150a	99	100	100	99	99	99	97	98
	EC-EBM-150b	100	100	99	99	98	97	97	97
	EC-EM-80	100	99	95	90	91	84	84	76
	EC-EM-100	99	100	95	95	95	90	90	88
	EC-EM-150	100	100	99	98	98	96	98	0
C specimen	C-EBM-40a	97	69	69	58	60	53	57	45
	C-EBM-40b	97	79	80	78	81	74	62	51
	C-EBM-60a	99	96	96	92	93	92	91	90
	C-EBM-60b	99	93	94	91	91	91	90	90
	C-EBM-80a	98	96	96	93	93	93	92	55
	C-EBM-80b	99	96	97	93	94	94	94	94
	C-EM-40	97	60	60	56	61	44	50	36
	C-EM-60	100	99	100	96	97	93	92	91
	C-EM-80	100	98	99	96	96	96	96	96
A specimen	E-EBM-120	98	99	98	98	97	97	98	98
	EC-EBM-120 ^(3)^	-	-	-	-	-	-	-	-
	C-EBM-100	100	98	99	97	98	96	98	97

^(1)^ Instantaneous TSP after applying load; ^(2)^ TSP value before increasing load; ^(3)^ FBG-OF sensor was broken during preparation process.

## Data Availability

Not applicable.

## Abbreviations and Acronyms

CN	Cyanoacrylate
DFOS	Distributed fiber optic sensors
EB	Externally bonded
EBM	External bonding method
EM	Embedded method
ESG	Electrical strain gauge
ESTC	Error in strain transfer coefficient
FBG	Fiber Bragg grating
FRP	Fiber-reinforced polymer
NSM	Near surface-mounted
OF	Optical fiber
PMMA	Polymethyl methacrylate
RC	Reinforced concrete
SEM	Scanning electron microscope
SHM	Structural health monitoring
STCE	Short-term creep effect
TSP	Transferred strain percentage
A	Additional
C	CN adhesive
E	Epoxy resin
EC	Epoxy resin and CN adhesive
